# Inhibition of rat locus coeruleus neurons by prostaglandin E_2_ EP3 receptors: pharmacological characterization *ex vivo*

**DOI:** 10.3389/fphar.2023.1290605

**Published:** 2023-11-15

**Authors:** Amaia Nazabal, Aitziber Mendiguren, Joseba Pineda

**Affiliations:** Department of Pharmacology, Faculty of Medicine and Nursing, University of the Basque Country (UPV/EHU), Leioa, Spain

**Keywords:** locus coeruleus, prostanoid EP3 receptor, prostaglandin E_2_, noradrenaline/norepinephrine, slice, electrophysiology, rat, firing

## Abstract

Prostaglandin E_2_ (PGE_2_) is an inflammatory mediator synthesized by the brain constitutive cyclooxygenase enzyme. PGE_2_ binds to G protein-coupled EP1-4 receptors (EP1 to G_q_, EP2,4 to G_s_, and EP3 to G_i/o_). EP2, EP3 and EP4 receptors are expressed in the locus coeruleus (LC), the main noradrenergic nucleus in the brain. EP3 receptors have been explored in the central nervous system, although its role regulating the locus coeruleus neuron activity has not been pharmacologically defined. Our aim was to characterize the function of EP3 receptors in neurons of the LC. Thus, we studied the effect of EP3 receptor agonists on the firing activity of LC cells in rat brain slices by single-unit extracellular electrophysiological techniques. The EP3 receptor agonist sulprostone (0.15 nM–1.28 µM), PGE_2_ (0.31 nM–10.2 µM) and the PGE_1_ analogue misoprostol (0.31 nM–2.56 µM) inhibited the firing rate of LC neurons in a concentration-dependent manner (EC_50_ = 15 nM, 110 nM, and 51 nM, respectively). The EP3 receptor antagonist L-798,106 (3–10 µM), but not the EP2 (PF-04418948, 3–10 µM) or EP4 (L-161,982, 3–10 µM) receptor antagonists, caused rightward shifts in the concentration-effect curves for the EP3 receptor agonists. Sulprostone-induced effect was attenuated by the G_i/o_ protein blocker pertussis toxin (pertussis toxin, 500 ng ml^-1^) and the inhibitors of inwardly rectifying potassium channels (GIRK) BaCl_2_ (300 µM) and SCH-23390 (15 µM). In conclusion, LC neuron firing activity is regulated by EP3 receptors, presumably by an inhibitory G_i/o_ protein- and GIRK-mediated mechanism.

## 1 Introduction

Prostaglandins are pain and inflammatory mediators synthesized on demand from membrane diacylglycerol, which is first converted into arachidonic acid via phospholipase-A2, then transformed to a common prostaglandin precursor by the rate-limiting enzyme cyclooxygenase (COX) and finally brought to the final product by different prostaglandin synthases. Nonsteroidal anti-inflammatory drugs (NSAIDs), widely used as analgesic, antipyretic and anti-inflammatory drugs, act by blocking COX and thereby suppressing the synthesis of prostaglandins. Brain prostaglandin E_2_ (PGE_2_) is a main final product of this pathway produced in endothelial cells and plays a role in inflammatory signaling after activation of astrocytes in the central nervous system (CNS). In neurons from specific brain areas, both COX-1 and COX-2 isoforms are also constitutively expressed under non-inflammatory conditions (Kaufmann et al., 1996; Hétu and Riendeau, 2005), supporting the idea that prostaglandins also play an essential role in regulating the function of the CNS. Thus, PGE_2_ modulates synaptic transmission in the hippocampus (Sang et al., 2005), the periaqueductal gray (PAG) (Lu et al., 2007) and the paraventricular nucleus of the hypothalamus (Khazaeipool et al., 2018). The variety of PGE_2_ actions are believed to be the result of four PGE_2_ receptor subtypes (EP1-4), which are coupled to different G protein pathways, primarily EP1 to G_q_, EP2 and EP4 to G_s_, and EP3 to G_i/o_. The EP2, EP3 and EP4 receptors are widely expressed in the CNS, including the hypothalamus and the locus coeruleus (LC) (Zhang and Rivest, 1999; Ek et al., 2000). Among EP receptors, the functional role of the EP3 receptor has been one of the most extensively studied in the brain. Thus, EP3 receptor activation depolarizes serotonergic neurons in the dorsal raphe nucleus (Momiyama et al., 1996), weakens the long-term potentiation in the hippocampus (Maingret et al., 2017) and mediates the response to hypercapnia in the chemosensitive parafacial respiratory group (Forsberg et al., 2016). EP3 receptors within the PAG facilitate spinal nociception in arthritic secondary hypersensitity (Drake et al., 2016). In addition, EP3 receptors have been shown to be involved in nociception transmission (Drake et al., 2016), febrile response and neuroinflammation in the central nervous system (Ushikubi et al., 1998; Almeida et al., 2004; Shi et al., 2012).

The LC, the main source of noradrenaline (NA) in the brain, is involved in the regulation of numerous central functions, such as pain, rewarding, arousal, sleep-wake cycle and cognition. LC neurons possess the enzymatic machinery to synthesize prostaglandins, including the constitutive COX-2 (Yamaguchi and Okada, 2009). Several pieces of evidence have associated the LC with the EP3 receptor. First, *in situ* hybridization and immunohistochemistry techniques have detected a high expression of the EP3 receptor subtype and its mRNA in the LC (Ek et al., 2000; Nak et al., 2001). Second, behavioral studies have demonstrated that administration of an EP3 receptor agonist reverses the naloxone-induced c-fos overexpression observed in the LC from morphine-dependent rats (Nakagawa et al., 2000). Finally, presynaptic EP3 receptors mediate the inhibition of NA release in two main projection areas of the LC, the cortex and the hippocampus (Exner and Schlicker, 1995; Günther et al., 2010).

A recent study in an transgenic mouse line has shown a female-specific enrichment of the EP3 receptor-encoding Ptger3 gene in the LC (Mulvey et al., 2018). Electrophysiological recordings with patch-clamp techniques in C57/BL6J mice have suggested that the EP3 receptor mediates the hyperpolarization of LC neurons induced by administration of a single, high concentration of the agonist sulprostone (Mulvey et al., 2018). However, it remains uncharacterized the full pharmacological profile of this prostanoid receptor subtype in wild-type rats, that is, the response of LC neurons to a wide range of agonist concentrations with or without different antagonists, the functional sensitivity of the receptor to different natural or synthetic prostaglandin analogs, and the molecular mechanism underlying the receptor-mediated effect in the LC. Therefore, to directly characterize the EP3 receptor and its signaling in this nucleus, we carried out single-unit extracellular recordings of LC neurons from rat brain slices and constructed whole concentration-effect curves for different EP3 receptor agonists in control and after various specific antagonists.

## 2 Materials and methods

### 2.1 Animals and ethics statement

87 adult male Sprague-Dawley rats (200–300 g) were used in this study. The animals were provided by the animal facilities of the University of the Basque Country (Leioa, Spain) and housed under standard environmental conditions (22°C, 12:12 h light/dark cycles) with free access to food and water. One slice was taken from each animal, and unless otherwise stated, only one experiment was performed in each slice. The number of experiments in each group was typically five to nine, depending on the level of variability. Treatment and control assays were performed in parallel in a randomized manner. As electrophysiological outcomes were collected *in situ*, data recording could not be blinded to the operator. However, the data analysis performed by the experimenter was confirmed separately by an additional researcher. All the experiments were carried out in compliance with the ARRIVE guidelines (Kilkenny et al., 2010) and according to EU Directive 2010/63 on the protection of animals used for scientific purposes. All the procedures were approved by the local Ethical Committee for Research and Teaching of the University of the Basque Country (UPV/EHU, Spain) and the Department of Sustainability and Natural Environment of Provincial Council from Bizkaia (ref. CEEA M20-2015-152; CEEA M20-2018-026). Every effort was made to use the minimum number of animals and reduce their suffering.

### 2.2 Brain slice preparation

Animals were anesthetized with chloral hydrate (400 mg kg^-1^, i.p.) and decapitated (Mendiguren and Pineda, 2007). The brain was rapidly extracted and a block of tissue including the brainstem was immersed in an ice-cold modified artificial cerebrospinal fluid (aCSF) where NaCl was equiosmolarly substituted for sucrose to improve neuronal viability. Coronal slices of 500–600 μm thickness containing the LC were cut using a vibratome (Leica VT1200 S, Leica Biosystems, Nussloch, Germany) (Paxinos and Watson, 2009). The slices were taken between interaural −0.60-0.96 mm and bregma −9.60-9.96 mm coordinates. This rostro-caudal section of the brainstem corresponds to the core of the LC, which is enriched with noradrenergic cell bodies according to previous ultrastructural analysis made with immunogold labelling (Van Bockstaele, 1998). The tissue was allowed to recover from the slicing for 90 min, placed on a nylon mesh and incubated at 33 ± 1 °C on a modified Haas-type interface chamber, which provided excellent perfusion to the slice. The tissue was continuously perfused with aCSF saturated with 95% O_2_/5% CO_2_ (final pH = 7.34) at a flow rate of 1.5 ml min^-1^ and left for equilibration before recordings were made. The aCSF contained (in mM): NaCl 130, KCl 3, NaH_2_PO_4_ 1.25, D-glucose 10, NaHCO_3_ 21, CaCl_2_ 2 and MgSO_4_ 2. The LC was recognized visually in the rostral pons as a dark oval area on the lateral borders of the central grey and the fourth ventricle, at or just anterior to the *genu* of the facial nerve. Previous histological analysis has verified that this area contains typical neurons of the LC showing noradrenergic cell properties (Williams et al., 1984).

### 2.3 Electrophysiological recordings

Single-unit extracellular recordings of LC noradrenergic cells were made as previously described (Mendiguren and Pineda, 2004; Mendiguren and Pineda, 2007). The recording electrode, an Omegadot glass micropipette, was pulled (Sutter Instruments, Novato, CA, USA) and filled with 50 mM NaCl. The tip of the electrode was broken back to a diameter of 2–5 μm (3–5 MΩ) and positioned in the core of the LC, which contains noradrenergic cell bodies. The extracellular signal from the electrode was passed through a high-input impedance amplifier (Axoclamp 2B, Molecular Devices, Union City, CA, USA) and monitored with an audio analyzer and also on an oscilloscope (Aumon 14, Cibertec S.A., Madrid, Spain). Individual neuronal spikes were isolated from the background noise with a window discriminator (PDV 225, Cibertec S.A.). The firing rate (FR) was continuously recorded and analyzed before, during and after experimental manipulations by a PC-based custom-made program which generated consecutive histogram bars representing the cumulative number of spikes in successive 10 s bins (HFCP^®^, Cibertec S.A., Madrid, Spain). Noradrenergic cells in the LC were identified by their spontaneous and regular discharge activities, the slow FR (0.5–1.5 Hz) and the long-lasting (3–4 ms) biphasic positive-negative waveforms (Mendiguren and Pineda, 2007).

### 2.4 Pharmacological procedures

The FR of LC neurons was recorded for several minutes before drug applications to obtain the baseline activity and then, during and after drug perfusion. We only used cells that showed stable FRs between 0.5 and 1.5 Hz for at least 3–5 min and clear inhibitory responses to control perfusion with [Met]enkephalin (ME, 0.8 μM, 1 min) or GABA (1 mM; 1 min) (inhibition magnitudes >80% of basal firing rate) (Pablos et al., 2015). To study the effect of EP3 receptor agonists on the firing rate of LC neurons, we perfused increasing concentrations of the EP3 receptor agonist sulprostone (0.15 nM–1.28 µM, 2x), PGE_2_ (0.31 nM–10.2 µM, 2x) or the PGE_1_ analogue misoprostol (0.31 nM–2.56 µM, 2x). Concentrations were based on previous studies in brain slices (Exner and Schlicker, 1995). To construct the concentration-effect curves, each concentration of the EP receptor agonists was perfused for enough time to reach its plateau effect (i.e., a steady firing rate in 3-4 consecutive 10-s bin bars), after which the following concentration (2x) was added up to concentrations that achieved the near-maximal cumulative effect of the drug. To identify the EP receptor subtype involved in the effects of sulprostone, PGE_2_ and misoprostol, the concentration-effect curves for these agonists were performed in the absence and the presence of different EP receptor subtype selective antagonists. Thus, in the case of sulprostone curves, we used the EP3 receptor antagonist L-798,106 (3 and 10 µM), the EP2 receptor antagonist PF-04418948 (3 and 10 µM) and the EP4 receptor antagonist L-161,982 (3 and 10 µM) at the concentrations previously reported (Jones et al., 2011). In the case of PGE_2_ and misoprostol curves, we used the EP3 receptor antagonist L-798,106 (10 µM) and a combination of the aforementioned EP2 and EP4 receptor antagonists (10 µM) at the maximal concentration. All the antagonists were perfused for 30 min before performing the concentration-effect curves for the EP receptor agonists.

Based on its pharmacological profile, we selected sulprostone to further characterize the molecular mechanism underlying EP3 receptor activation. In order to study the involvement of Gi/o proteins, concentration-effect curves for sulprostone were made after overnight incubation of the slices with the irreversible G_i/o_ protein inhibitor pertussis toxin (PTX, 500 ng ml^-1^, 18 h) in an oxygenated glass beaker at room temperature (modified from Chessell et al., 1996). In order to verify that PTX had effectively blocked the G_i/o_ protein, the effect of the G_i/o_-protein coupled MOR agonist ME (0.8 μM, 1 min) was tested. Thus, only cells with a blunted inhibitory effect of ME (that is, inhibition magnitudes weaker than 75% of basal FR) were considered to perform the concentration-effect curve for sulprostone. In those cells, proper drug perfusion was assessed with GABA (1 mM, 1 min) (that is, inhibition magnitudes greater than 80% of basal FR). To confirm the involvement of inwardly rectifying potassium channels (GIRK), we performed concentration-effect curves for sulprostone in the presence of the GIRK blocker BaCl_2_ (300 μM, 15 min) or the selective GIRK2 gating inhibitor SCH-23390 (15 μM, 30 min) at the concentrations previously used (Pineda and Aghajanian, 1997; Chee et al., 2011).

### 2.5 Analysis and statistics of electrophysiological data

The data and statistical analyses were carried out with the computer programs GraphPad Prism (version 5.0 for Windows, GraphPad Software, Inc., San Diego, CA, USA) and SPSS (version 22.0 for Windows, SPSS Inc., Chicago, IL, USA); these procedures comply with the recommendations on experimental design and analysis in pharmacology (Curtis et al., 2018). To obtain the experimental data of the concentration-effect curves for EP3 receptor agonists, the cumulative inhibitory effects of sulprostone, PGE_2_ or misoprostol were first normalized to the baseline FR in each cell as follows: *E (%) =* (*FR*_*basal*_ *- FR*_*post*_) *· 100/FR*_*basal*_, where *FR*_*basal*_ is the spontaneous FR of each neuron averaged for 60 s immediately before the first agonist concentration and *FR*_*post*_ is the FR of the same neuron averaged for 60–90 s after each drug application. This post-drug interval was chosen to integrate the whole period of maximal effect of each drug administration (Medrano et al., 2017). Normalization to baseline values was made to obtain comparable measures across and within groups.

To construct the theoretical concentration-effect curves for the EP3 receptor agonists, we performed nonlinear analysis of experimental curve data and obtained the best simple curve fit values to the following three-parameter logistic equation: E = E_max*·*_ A^*n*^/(A^*n*^ + EC_50_^*n*^), where *E* and *A* are the observed cumulative effect and the concentration of the agonist, respectively; *E*_max_ is the maximal effect of the EP3 receptor agonist with the E_max_ being constrained to 100% in those neurons in which the observed effect reached a full cessation of the FR. *EC*_*50*_ is the concentration of the agonist required to promote the 50% of E_max_ and *n* represents the slope factor of the function. These parameters were individually determined by the nonlinear analysis and then averaged to obtain the theoretical parameters in each group. For comparison purposes, the EC_50_ values were converted and expressed as negative logarithm (pEC_50_) to transform the variable into a Gaussian distribution (Pineda et al., 1997a). The antagonist affinity value was calculated as the negative logarithm of the equilibrium dissociation constant of the antagonist-receptor complex (*pK*_*B*_). The pK_B_ value was estimated by nonlinear regression analysis of the entire concentration-effect curve after the antagonist by directly fitting the individual curve to the following equation derived from Schild model (Waud et al., 1978): E = E_max*·*_ A^*n*^/{A^*n*^ + [EC_50*·*_ (1 + (B/K_B_)^s^)]^*n*^}, where *E* and *A* are the observed effects and the agonist concentrations in the presence of a fixed concentration of the antagonist (*B*); *E*_max_, *EC*_*50*_ and *n* are the parameters of the concentration-effect curves for the agonist in the absence of the antagonist, which were constrained to the average values previously calculated in control; and *s* is the logistic slope factor of the antagonist occupancy function (fixed to 1 in accordance with reports by Lazareno et al., 1993 and Säfholm et al., 2013). This procedure has the advantage that all the curve data are used to estimate the antagonist affinity and, therefore, it yields more accurate estimates of these parameters than using just a few dose-ratio values by Schild analysis (Lazareno et al., 1993; Säfholm et al., 2013).

Data are expressed as the mean ± SEM of *n* number of experiments. Statistical analyses were performed by a two-tailed paired Student’s t-test when the response values were compared before and after drug applications within the same cell, and by a two-tailed two-sample Student’s t-test when the FRs, responses or parameters were compared under two independent experimental conditions. Statistical comparison of the results among more than two experimental conditions (including the control group) were performed by one-way analysis of variance (ANOVA) followed by *post hoc* pairwise comparisons with the Dunnett’s test or the Bonferroni’s Multiple Comparison test, provided that *F* achieved the necessary level of statistical significance (i.e., *p <* 0.05) and there was no significant variance in homogeneity. Dunnett’s procedure was used for comparisons with a control group and Bonferroni’s test, for comparisons among all the groups. The threshold of significance was set at *p* = 0.05 and only one level of probability (*p* < 0.05) is reported.

### 2.6 Materials and drugs

For electrophysiological recordings, the following drugs were used (drug source): BaCl_2_ (Sigma-Aldrich Química S.A., Madrid, Spain), γ-aminobutyric acid (GABA, Sigma-Aldrich), L-161,982 (Tocris Bioscience, Bristol, UK), L-798,106 (Tocris Bioscience), [Met]enkephaline acetate salt (Bachem, Weil am Rhein, Germany), misoprostol free acid (Cayman Chemical, Ann Arbor, MI, USA), pertussis toxin (Tocris Bioscience), PF-04418948 (Tocris Bioscience), prostaglandin E_2_ (PGE_2_) (Tocris Bioscience), SCH-23390 (Tocris Bioscience) and sulprostone (Cayman Chemical). Stock solutions of L-161,982, L-798,106, PF-04418948 and PGE_2_ were first prepared in pure DMSO and then diluted in aCSF to obtain a final concentration of DMSO lower than 0.1%, which does not affect the firing activity of LC neurons *ex vivo* (Pineda et al., 1996). Misoprostol and sulprostone were purchased already dissolved in methyl acetate, and the final concentrations were obtained by diluting them in the aCSF at the moment of the experiment. Control assays were performed with equivalent volumes of the vehicles in which the drugs were dissolved. The final concentration of methyl acetate in the aCSF was <0.01%. Stock solutions of the rest of the drugs were first prepared in Milli-Q water and then diluted 1000 to 10000 fold in aCSF for the desired concentration. Final solutions were freshly prepared just before each experiment and stock solutions were kept at −20°C.

## 3 Results

### 3.1 Pharmacological characterization of the effect of sulprostone, a selective EP3 receptor agonist, on the firing rate of LC neurons

**FIGURE 1:**
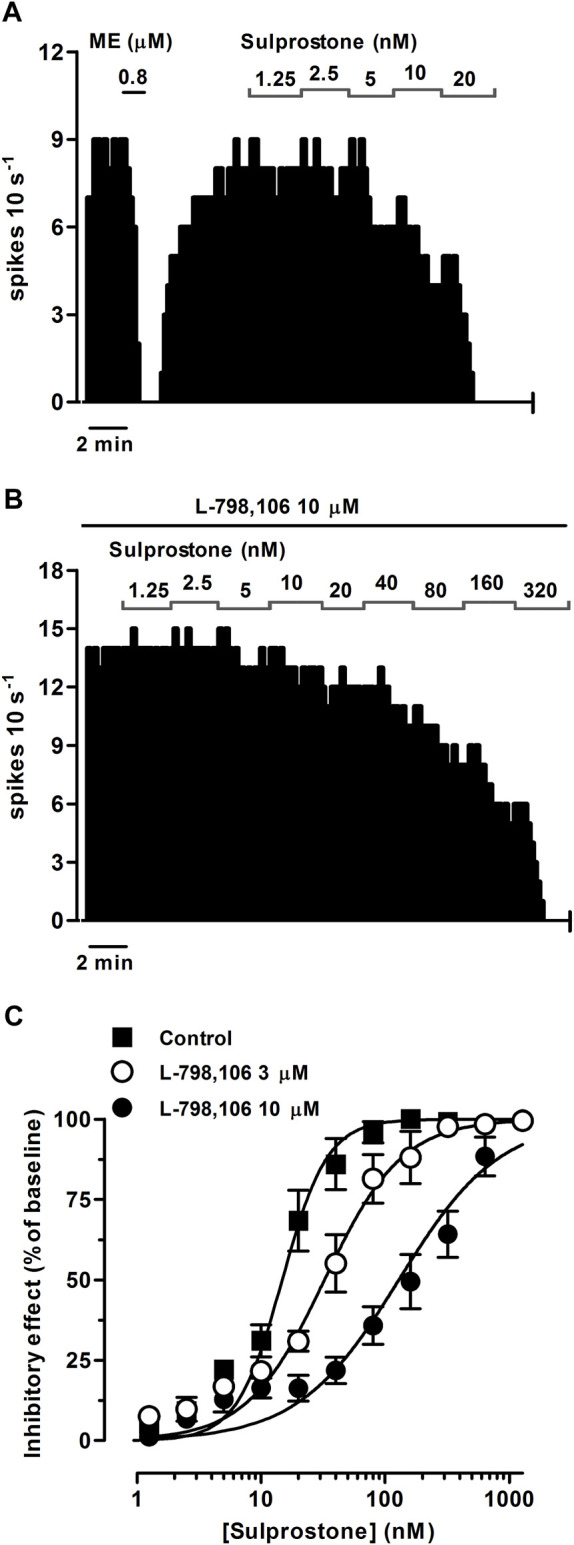
Effect of the EP3 receptor agonist sulprostone on the firing rate of LC neurons in the absence or presence of the EP3 receptor antagonist L-798,106. **(A,B)** Representative examples of firing rate recordings of two LC neurons showing the effect of increasing concentrations of sulprostone in the absence **(A)** and presence of L-798,106 (10 µM) **(B)**. The vertical lines represent the number of spikes recorded every 10 s and the horizontal bars the period of drug application. **(C)** Concentration-effect curves for sulprostone in control (filled squares) and in the presence of L-798,106 (3 μM, open circles or 10 μM, filled circles). The horizontal axis shows the sulprostone concentration on a semi-logarithmic scale. The vertical axis expresses the reduction in firing rate of LC neurons as the percentage of the baseline. Data points are the mean ± SEM at each sulprostone concentration obtained from *n* number of experiments. The lines through the data are the theoretical curves in each group constructed from the mean of the individual concentration-effect curve parameters, as estimated by nonlinear regressions. Note that the concentration-effect curve for sulprostone is shifted to the right by the EP3 receptor antagonist.

EP3 receptors are expressed in catecholaminergic neurons of the brain, including the LC (Yamaguchi and Okada, 2009). To investigate the regulation mediated by EP3 receptors in the firing rate of LC neurons, we performed concentration-effect curves for sulprostone, an agonist that shows more than 300-fold higher affinity for the EP3 than the EP1 receptor (Abramovitz et al., 2000). Perfusion with sulprostone (1.25–320 nM, 2x, 1 min each) inhibited the firing activity of LC neurons in a concentration-dependent manner, with an EC_50_ in the nanomolar range (Figures 1A, C; Table 1). Complete inhibition of the firing rate of noradrenergic cells was achieved at the highest concentrations of sulprostone (≥ 20 nM) and persisted for 265 ± 53 s on average (*n* = 9). A recovery of more than 75% of the basal firing rate was observed after 11 min of the complete inhibition produced by sulprostone (*n* = 3).

**TABLE 1:** Basal firing rate and concentration-effect curve parameters for the inhibitory action of the EP3 receptor agonist sulprostone on LC neurons in the absence (control) or in the presence of the EP3 (L-798,106), EP2 (PF-04418948), and EP4 (L-161,982) receptor antagonists.

Concentration-effect curves^a^						
Drugs	Concentration (µM)	Basal firing rate (Hz)	pEC_50_ (M)	(EC_50_, nM)	Slope factor	*n*
Sulprostone						
Control		0.84 ± 0.07	7.83 ± 0.08	(14.8)	2.39 ± 0.37	9
+ L-798,106	3	0.64 ± 0.11	7.49 ± 0.09	(32.4)	1.39 ± 0.16*	6
	10	0.87 ± 0.22	6.89 ± 0.11*	(128)	1.07 ± 0.12*	6
+ PF-04418948	3	0.66 ± 0.11	7.63 ± 0.15	(23.5)	1.89 ± 0.14	6
	10	0.58 ± 0.08	8.49 ± 0.10*	(3.25)	1.61 ± 0.21	5
+ L-161,982	3	0.64 ± 0.11	8.05 ± 0.11	(8.93)	1.92 ± 0.36	5
	10	0.71 ± 0.12	8.08 ± 0.11	(8.34)	1.49 ± 0.11	5

^a^ Values are expressed as mean ± SEM, obtained by nonlinear regression of *n* cells. Maximal effect values were 100% in all cases. pEC_50_ is the negative logarithm of the concentration needed to elicit 50% of the maximal effect. *p < 0.05 when compared to the control group (one-way ANOVA, followed by a Dunnett’s *post hoc* test).

**FIGURE 2:**
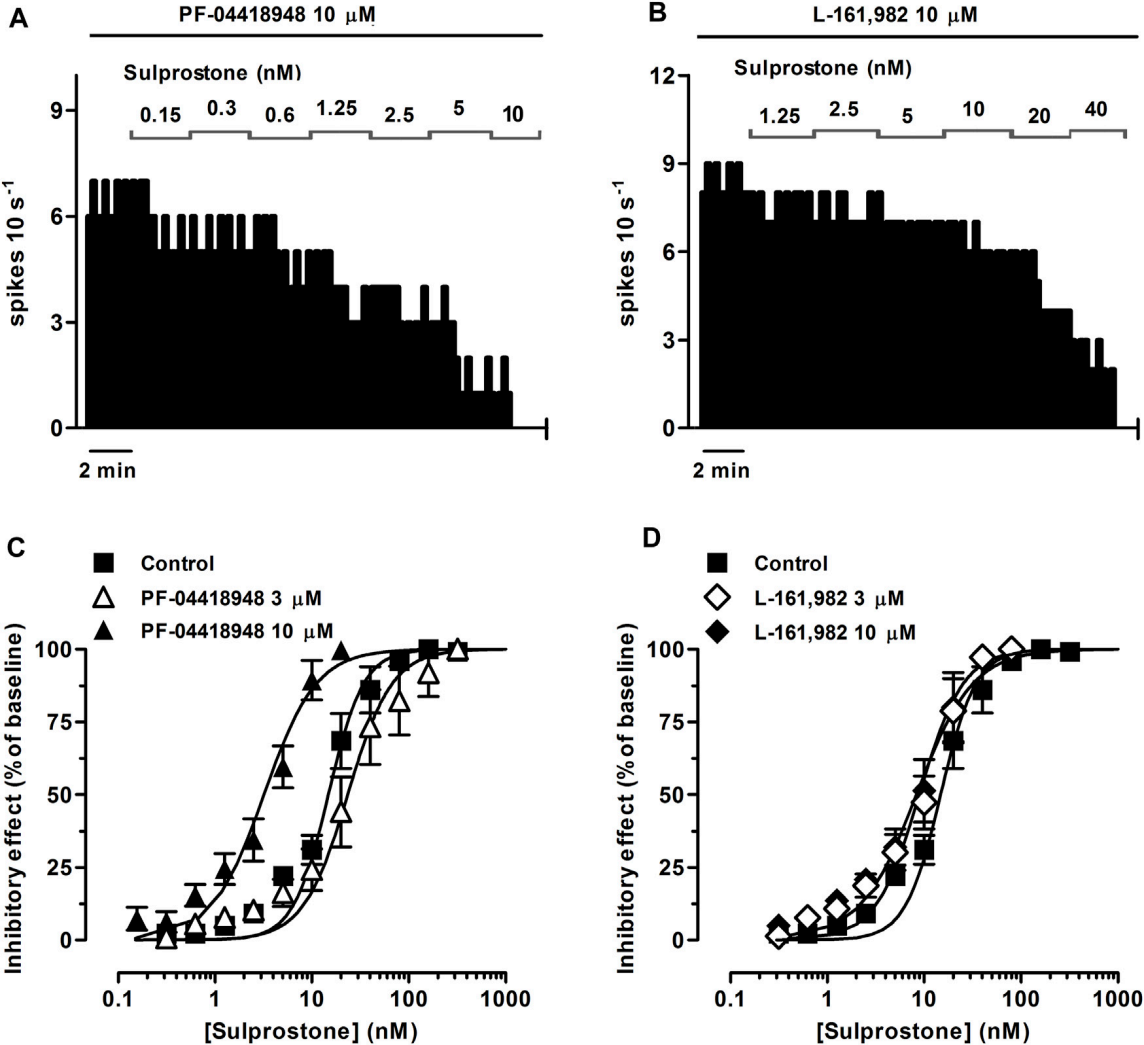
Effect of the EP3 receptor agonist sulprostone on the firing rate of LC neurons in the presence of the EP2 receptor antagonist PF-04418948 or the EP4 receptor antagonist L-161,982. **(A,B)** Representative examples of firing rate recordings of two LC neurons showing the effect of increasing concentrations of sulprostone in the presence of PF-04418948 (10 µM) **(A)** or L-161,982 (10 µM) **(B)**. The vertical lines represent the number of spikes recorded every 10 s and the horizontal bars the period of drug application. **(C,D)** Concentration-effect curves for sulprostone in control (filled squares) and in the presence of PF-04418948 (3 μM, open triangles or 10 μM, filled triangles) **(C)** or L-161,982 (3 μM, open diamonds or 10 μM, filled diamonds) **(D)**. The horizontal axes show the sulprostone concentration on a semi-logarithmic scale. The vertical axes express the reduction in firing rate of LC neurons as the percentage of the baseline. Data points are the mean ± SEM at each sulprostone concentration obtained from *n* number of experiments. The lines through the data are the theoretical curves in each group constructed from the mean of the individual concentration-effect curve parameters, as estimated by nonlinear regressions.

In order to investigate the EP receptor involved in the sulprostone-induced inhibitory effect, concentration-effect curves for sulprostone (0.15 nM–1.28 µM) were performed in the presence of the EP3 receptor antagonist L-798,106 (3 and 10 µM), the EP2 receptor antagonist PF-04418948 (3 and 10 µM) or the EP4 receptor antagonist L-161,982 (3 and 10 µM). Thus, perfusion with L-798,106 (3 µM) for 30 min did not significantly change the firing rate of LC neurons, but shifted to the right the concentration-effect curve for sulprostone, with a 2.2-fold increase in the EC_50_ and a significant reduction in the steepness of the curves (Figure 1C; Table 1). Moreover, a higher concentration of L-798,106 (10 μM, 10 min) reduced the firing rate of LC neurons by 17.7% ± 3.8% (*n* = 6, *p* < 0.05 *vs* baseline in the same neuron) and shifted to the right by 8.6 fold the concentration-effect curve for sulprostone (Figures 1B, C; Table 1). The estimated apparent affinity of L-798,106 for the EP3 receptor (pK_B_) was 5.77 ± 0.10 (*n* = 12). On the other hand, the EP2 receptor antagonist PF-04418948 (3 µM) failed to cause any significant change in the firing rate or the concentration-effect curve for sulprostone (Figure 2C; Table 1). Unexpectedly, the highest concentration of PF-04418938 (10 µM) caused a 4.6-fold shift to the left in the concentration-effect curve for sulprostone (Figures 2A, C; Table 1). Finally, perfusion with the EP4 receptor antagonist L-161,982 (3 and 10 µM) failed to change the firing rate or the concentration-effect curves for sulprostone (Figures 2B, D; Table 1).

Altogether, these results suggest that the inhibitory effect of sulprostone on the firing rate of LC neurons is mediated by the EP3 receptor.

### 3.2 Pharmacological characterization of the effects of PGE_2_ and misoprostol, two mixed EP3 receptor agonists, on the firing rate of LC neurons

**FIGURE 3:**
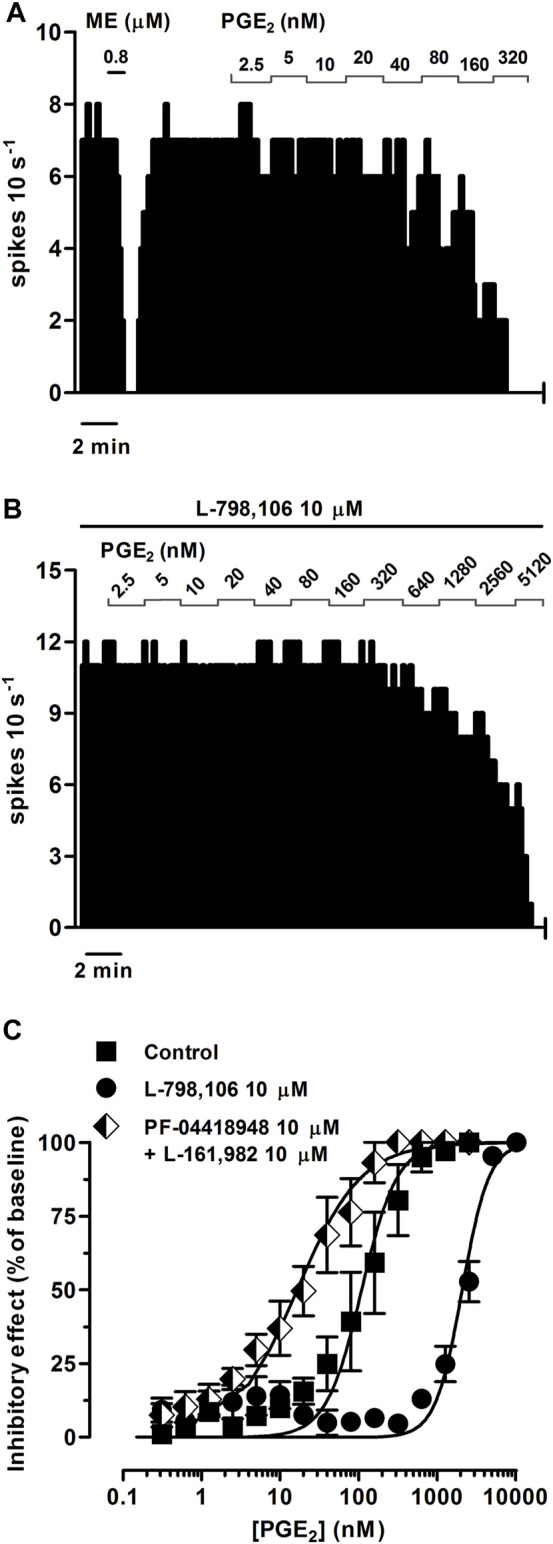
Effect of PGE_2_ on the firing rate of LC neurons in the absence or presence of the EP3 receptor antagonist L-798,106 or a combination of the EP2 receptor antagonist PF-04418948 and the EP4 receptor antagonist L-161,982. **(A,B)** Representative examples of firing rate recordings of two LC neurons showing the effect of increasing concentrations of PGE_2_ in the absence **(A)** and presence of L-798,106 (10 µM) **(B)**. The vertical lines represent the number of spikes recorded every 10 s and the horizontal bars the period of drug application. **(C)** Concentration-effect curves for PGE_2_ in control (filled squares) and in the presence of L-798,106 (10 μM, filled circles) or PF-04418948 and L-161,982 (10 µM each, half-filled diamonds). The horizontal axis shows the PGE_2_ concentration on a semi-logarithmic scale. The vertical axis expresses the reduction in firing rate of LC neurons as the percentage of the baseline. Data points are the mean ± SEM at each PGE_2_ concentration obtained from *n* number of experiments. The lines through the data are the theoretical curves in each group constructed from the mean of the individual concentration-effect curve parameters, as estimated by nonlinear regressions. Note that the concentration-effect curve for PGE_2_ is shifted to the right by the EP3 receptor antagonist and to the left by the EP2 and EP4 receptor antagonists.

**FIGURE 4:**
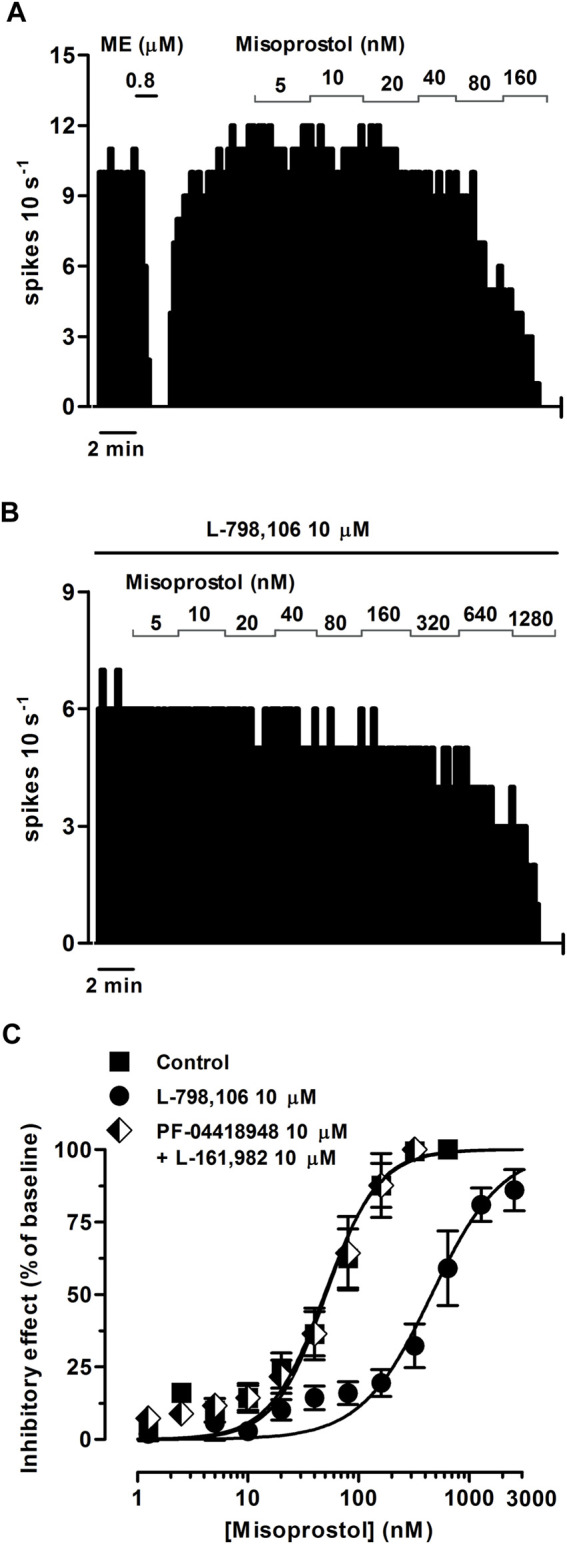
Effect of misoprostol on the firing rate of LC neurons in the absence or presence of the EP3 receptor antagonist L-798,106 or a combination of the EP2 receptor antagonist PF-04418948 and the EP4 receptor antagonist L-161,982. **(A,B)** Representative examples of firing rate recordings of two LC neurons showing the effect of increasing concentrations of misoprostol in the absence **(A)** and presence of L-798,106 (10 µM) **(B)**. The vertical lines represent the number of spikes recorded every 10 s and the horizontal bars the period of drug application. **(C)** Concentration-effect curves for misoprostol in control (filled squares) and in the presence of L-798,106 (10 μM, filled circles) or PF-04418948 and L-161,982 (10 µM each, half-filled diamonds). The horizontal axis shows the misoprostol concentration on a semi-logarithmic scale. The vertical axis expresses the reduction in firing rate of LC neurons as the percentage of the baseline. Data points are the mean ± SEM at each misoprostol concentration obtained from *n* number of experiments. The lines through the data are the theoretical curves in each group constructed from the mean of the individual concentration-effect curve parameters, as estimated by nonlinear regressions. Note that the concentration-effect curve for misoprostol is shifted to the right by the EP3 receptor antagonist.

To study whether PGE_2_ and the PGE_1_ analog misoprostol elicit EP3 receptor-mediated inhibitory effects on the firing rate of LC cells, we performed concentration-effect curves for both agonists. PGE_2_ (0.31 nM–1.28 µM, 2x, 1 min each) and misoprostol (0.31–320 nM, 2x, 1 min each) both inhibited the firing rate of LC neurons in a concentration-dependent manner, with EC_50_ values in the intermediate nanomolar range (Figures 3A, C; Figures 4A, C; Table 2).

**TABLE 2:** Basal firing rate and concentration-effect curve parameters for the inhibitory action of the EP3 receptor agonists PGE_2_ and misoprostol on LC neurons in the absence (control) or in the presence of the EP3 (L-798,106) or a combination of the EP2 (PF-04418948) and EP4 (L-161,982) receptor antagonists.

Concentration-effect curves^a^						
Drugs	Concentration (µM)	Basal firing rate (Hz)	pEC_50_ (M)	(EC_50_, nM)	Slope factor	*n*
PGE2						
Control		0.76 ± 0.10	6.96 ± 0.20	(110)	1.92 ± 0.36	5
+ L-798,106	10	0.87 ± 0.14	5.68 ± 0.05*	(2098)	2.55 ± 0.47	5
+ PF-04418948	10					
L-161,982	10	0.83 ± 0.24	7.78 ± 0.19*	(16.6)	1.07 ± 0.06	5
Misoprostol						
Control		0.80 ± 0.12	7.30 ± 0.13	(50.7)	1.85 ± 0.30	5
+ L-798,106	10	0.67 ± 0.11	6.34 ± 0.12*	(455)	1.36 ± 0.28	5
+ PF-04418948	10					
L-161,982	10	0.72 ± 0.11	7.31 ± 0.12	(49.5)	1.76 ± 0.33	5

^a^ Values are expressed as mean ± SEM, obtained by nonlinear regression of *n* cells. Maximal effect values were 100% in all cases. pEC_50_ is the negative logarithm of the concentration needed to elicit 50% of the maximal effect. *p < 0.05 when compared to their respective control group (one-way ANOVA, followed by a Dunnett’s *post hoc* test).

Perfusion with the EP3 receptor antagonist L-798,106 (10 µM) shifted to the right by 19.1 and 9.0 fold the concentration-effect curves for PGE_2_ (0.31 nM–10.2 µM) and misoprostol (0.31 nM–2.56 µM), respectively (Figures 3B, C; Figures 4B, C; Table 2). The antagonist affinity for L-798,106 was higher when estimated with PGE_2_ as the agonist (pK_B_ = 6.26 ± 0.05, *n* = 5, *p* < 0.05) than the previous value estimated with sulprostone (see above). However, the antagonist affinity for L-798,106 estimated with misoprostol (pK_B_ = 5.91 ± 0.14; *n* = 5, *p* > 0.05) was not different from that obtained with sulprostone. On the other hand, administration of a combination of the EP2 receptor antagonist PF-04418948 (10 µM) and the EP4 receptor antagonist L-161,982 (10 µM) caused a 6.6-fold shift to the left in the concentration-effect curves for PGE_2_ (Figure 3C; Table 2). This combination of antagonists (PF-04418948 10 µM + L-161,982 10 µM) did not cause any significant shift in the concentration-effect curves for misoprostol (Figure 4C; Table 2). Finally, none of these antagonists changed the slope of the concentration-effect curves.

As a whole, these results suggest that the natural prostanoid PGE_2_ and the PGE_1_ synthetic analog misoprostol inhibit the activity of LC neurons through the EP3 receptor.

### 3.3 Molecular mechanisms underlying the EP3 receptor-mediated effect on the firing rate of LC neurons

**FIGURE 5:**
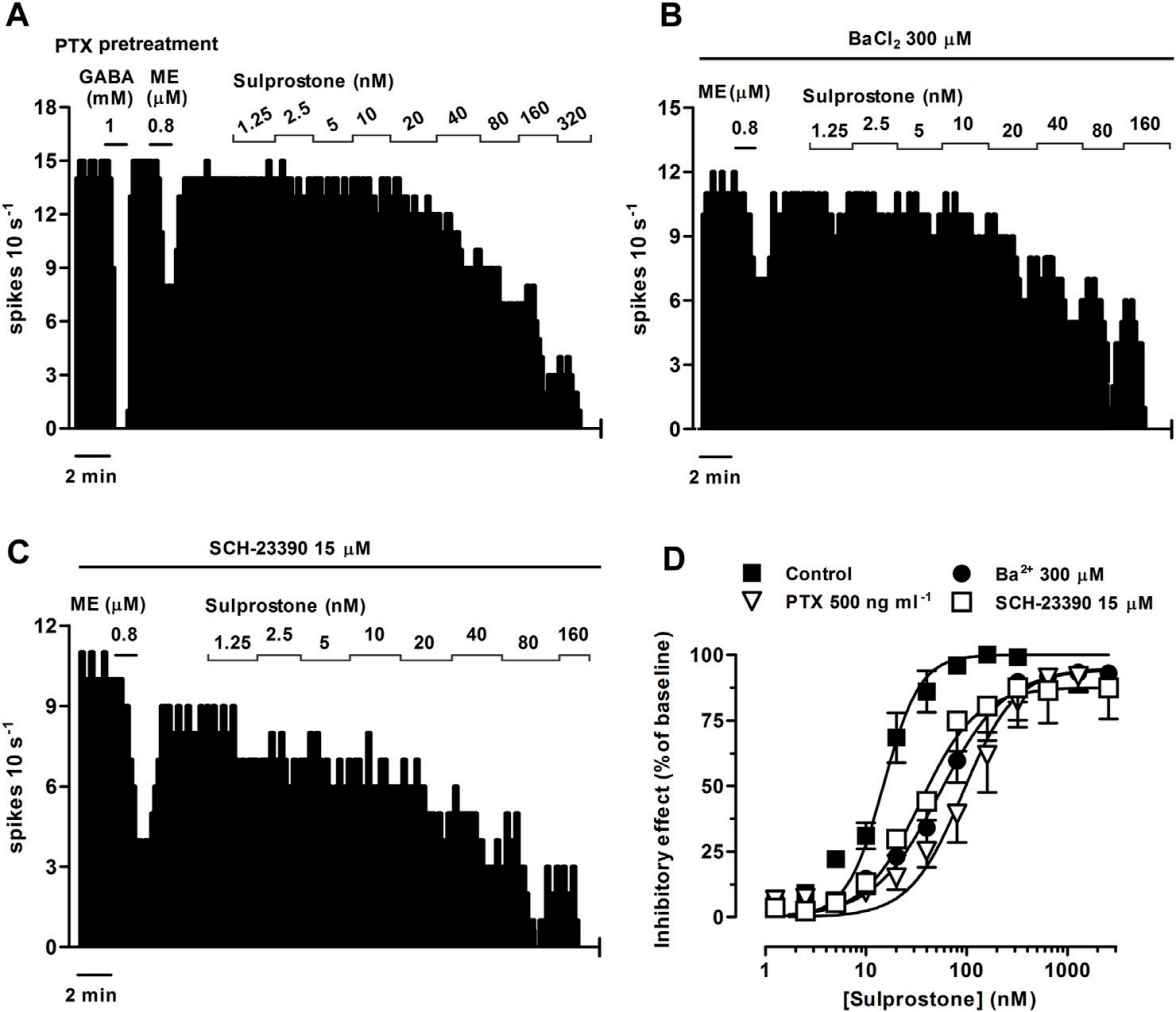
Effect of sulprostone on the firing rate of LC neurons after overnight treatment of the slices with the G_i/o_ inhibitor pertussis toxin (PTX) or in the presence of the GIRK blockers Ba^2+^ or SCH-23390. Representative examples of firing rate recordings of three LC neurons showing the effect of increasing concentrations of sulprostone after overnight treatment with PTX (500 ng ml^-1^) **(A)** or in the presence of BaCl_2_ (300 µM) **(B)** or SCH-23390 (15 µM) **(C)**. The vertical lines represent the number of spikes recorded every 10 s and the horizontal bars the period of drug application. Note that the effect of ME (0.8 µM) is reduced compared to control (see Figure 1A), while the inhibitory effect of GABA (1 mM) is maintained **(A)**. **(D)** Concentration-effect curves for sulprostone in control (filled squares) or after overnight treatment with PTX (500 ng ml^-1^, open triangles) or in the presence of Ba^2+^ (300 μM, filled circles) or SCH-23390 (15 μM, open squares). The vertical axis expresses the reduction in firing rate of LC as the percentage of the baseline. Data points are the mean ± SEM at each sulprostone concentration obtained from *n* number of experiments. The lines through the data are the theoretical curves in each group constructed from the mean of the individual concentration-effect curve parameters, as estimated by nonlinear regressions. Note that the concentration-effect curve for sulprostone is shifted to the right by PTX, Ba^2+^, and SCH-23390.

The EP3 receptor has been shown to be coupled to G_i/o_ proteins (Ikeda-Matsuo et al., 2010) and GIRK channels (Ruiz-Velasco and Ikeda, 1998). Thus, to identify the molecular mechanisms underlying the EP3 receptor-mediated inhibition of the firing activity of LC cells, we performed concentration-effect curves for sulprostone (0.31 nM–1.28 µM, 2x) in slices continuously incubated for 18 h with the irreversible G_i/o_ protein blocker PTX (500 ng ml^-1^). As a control for effective blockade of G_i/o_ proteins, concentration-effect curves for sulprostone were performed only in slices in which a reduction of the inhibitory response to the G_i/o_-coupled MOR agonist ME (0.8 µM, 1 min) had been previously confirmed after incubation with PTX (i.e., ME-induced inhibition <75% of basal firing rate; mean inhibition = 47.0 ± 13.7%, *n* = 5) (Figure 5A). In these cases, proper drug perfusion was tested with GABA (1 mM, 1 min) (Figure 5A), which is known to fully inhibit the firing rate of LC cells through GABA_A_ ionotropic receptors (i.e., GABA-induced inhibition >80% of basal firing rate). Thus, overnight treatment of the slices with PTX shifted to the right by 6.1 fold the concentration-effect curves for sulprostone, without affecting significantly the maximal response or the basal firing rates (Figures 5A, D; Table 3).

**TABLE 3:** Basal firing rate and concentration-effect curve parameters for the inhibitory action of sulprostone on LC neurons in the absence (control) or in the presence of PTX, BaCl_2_, and SCH-23390.

Concentration-effect curves^a^							
Drugs	Concentration	Basal firing rate (Hz)	Emax (%)	pEC_50_ (M)	(EC_50_, nM)	Slope factor	*n*
Sulprostone							
Control		0.84 ± 0.07	100	7.83 ± 0.08	(14.8)	2.39 ± 0.37	9
+ PTX	500 ng ml^-1^	1.12 ± 0.18	94.9	7.04 ± 0.15*	(90.9)	1.65 ± 0.28	5
+ BaCl_2_	300 µM	1.03 ± 0.13	94.6	7.27 ± 0.09*	(53.7)	1.37 ± 0.18	5
+ SCH-23390	15 µM	1.37 ± 0.28	87.5	7.46 ± 0.05*	(34.6)	1.56 ± 0.17	5

^a^ Values are expressed as mean ± SEM, obtained by nonlinear regression of *n* cells. Emax is the maximal inhibitory effect and pEC_50_ is the negative logarithm of the concentration needed to elicit 50% of the Emax. *p < 0.05 when compared to the control group (one-way ANOVA, followed by a Dunnett’s *post hoc* test).

To study the involvement of GIRK channels, we performed concentration-effect curves for sulprostone (0.31 nM–2.56 µM, 2x) in the presence of the non-selective GIRK channel blocker BaCl_2_ or the selective GIRK2 gating inhibitor SCH-23390 (Torrecilla et al., 2002). As a control for effective blockade of GIRK channels, we confirmed the reduction in the inhibitory response to the GIRK-coupled MOR agonist ME (0.8 µM, 1 min) by incubation with Ba^2+^ (300 μM, 15 min) and SCH-23390 (15 μM, 30 min) before the concentration-effect curves for sulprostone (i.e., mean ME-induced inhibition, 62.4 ± 7.0%, *n* = 5, *p* < 0.05 *vs* pre-blocker; and 71.8 ± 7.8%, *n* = 5; *p* < 0.05 *vs* pre-blocker; respectively) (Figures 5B, C). Bath perfusion with BaCl_2_ (300 μM, 15 min) increased the firing rate of LC neurons by 42.6 ± 13.1% (*n* = 5, *p* < 0.05 compared to baseline) and shifted to the right by 3.6 fold the concentration-effect curve for sulprostone (Figures 5B, D; Table 3). Likewise, bath administration of SCH-23390 (15 μM, 30 min) increased the firing activity of LC neurons by 144 ± 57% (*n* = 5, *p* < 0.05 compared to baseline) and shifted to the right by 2.3 fold the concentration-effect curve for sulprostone (Figures 5C, D; Table 3). BaCl_2_ and SCH-23390 slightly reduced the maximal effects of sulprostone, but these changes did not reach statistical significances (Table 3).

These results suggest that EP3 receptor activation inhibits the LC neuron activity through activation of G_i/o_ proteins and GIRK channels.

## 4 Discussion

Previous assays have shown that administration of a single, high concentration of an EP3 receptor agonist causes a hyperpolarizing response in mice LC neurons (Mulvey et al., 2018). The present work was undertaken to directly characterize, by single-unit extracellular recordings, the pharmacological nature and underlying mechanism of functional EP3 receptors in rat LC neurons. We constructed whole concentration-effect curves for different EP3 receptor agonists after perfusion with selective EP2/3/4 receptor antagonists in the LC from brain slices. Our results show that the selective EP3 receptor agonist sulprostone and the mixed EP3 receptor agonists PGE_2_ and misoprostol all induce concentration-dependent inhibitions of the neuronal firing activity of LC cells. The inhibitory effects were blocked by the selective EP3 receptor antagonist L-798,106, but not by the EP2 receptor antagonist PF-04418948 or the EP4 receptor antagonist L-161,982, which indicates that the effects of these agonists were mediated by EP3 receptors. Furthermore, sulprostone-induced inhibition of the firing activity was prevented by the G_i/o_ protein inhibitor PTX or the GIRK blockers BaCl_2_ and SCH-23390. Thus, our results support that pharmacologically identified EP3 receptors in LC neurons mediate the inhibitory regulation of firing activity by prostaglandin analogs through its coupling to G_i/o_ proteins and the subsequent opening of GIRK channels.

Activation of LC neurons by pro-inflammatory and nociceptive-like stimuli depends on brain PGE_2_ synthesis by the rate-limiting enzyme COX and the microsomal PGE synthase 1 (Hall et al., 1999; Dallaporta et al., 2007). Accordingly, COX isoenzymes have been reported to be constituvely expressed in spinally projecting LC neurons (Nak et al., 2001). Regarding the functional targets for prostaglandins, double immunofluorescence techniques have shown a dense EP3 receptor-like reactivity in the neuropil and cell bodies of noradrenergic neurons in the LC (Yamaguchi and Okada, 2009). Furthermore, a strong expression of the EP3 receptor gene (Mulvey et al., 2018) and mRNA (Ek et al., 2000) is present in the LC. In fact, intensive hybridization signal for EP3 receptor mRNA is specifically localized to neurons within the LC (Sugimoto et al., 1994). In our study, we assessed the functional role of EP3 receptors by selective and mixed EP3 receptor agonists combined with extracellular electrophysiological recording techniques *ex vivo*. Sulprostone was selected as a selective EP3 receptor agonist, since it has more than 300-fold higher affinity for EP3 than EP1 receptors (Abramovitz et al., 2000). Sulprostone was perfused at similar concentrations to those used to study the functional role of EP3 receptors in the cortex from rodent brain slices (Exner and Schlicker, 1995). We also assessed the biological relevance of the prostanoid system by perfusing PGE_2_, a natural prostaglandin showing high affinity for all the EP receptors, mainly for the EP3 receptor (EP3≥EP4>EP2>EP1) (Abramovitz et al., 2000). Moreover, we tested the commercially available PGE_1_ analog misoprostol, which is a preferential agonist of the EP3 receptor (affinity: EP3>EP4>EP2) (Abramovitz et al., 2000). On the other hand, the pharmacological nature of agonist effects was evaluated by the selective EP3 receptor antagonist L-798,106, which has more than 9000-fold higher affinity for EP3 than EP4 or EP2 receptors (Su et al., 2008). Finally, we examined the effect of the EP2 receptor antagonist PF-04418948, which displays more than 2000-fold higher selectivity for EP2 than EP3 or EP4 receptors (Forselles et al., 2011), and the effect of the EP4 receptor antagonist L-161,982, which shows 80 to 800-fold higher affinity for EP4 than EP3 or EP2 receptors (Machwate et al., 2001).

In the present study, the three EP3 receptor agonists sulprostone, PGE_2_ and misoprostol were fully efficacious at inhibiting the firing activity of LC neurons. The rank order of EC_50_ values estimated from the concentration-effect curves for neuron firing inhibition induced by these agonists was sulprostone > misoprostol > PGE_2_ (EC_50_ values: 14.8, 50.7 and 110 nM, respectively). This pharmacological profile in the LC is highly concordant with the values obtained from rodent slices for an EP3 receptor subtype in the brain cortex, where sulprostone, misoprostol and PGE_2_ inhibit the NA release with potencies in the same range (EC_50_ values: 6.0, 10.0 and 18.2 nM, respectively) (Exner and Schlicker, 1995). In guinea-pig aorta, a comparable EC_50_ value has been also found for sulprostone-induced vasoconstriction (23 nM) (Jones et al., 1998).

On the other hand, the inhibitory effects induced by sulprostone, misoprostol and PGE_2_ were prevented by the selective EP3 receptor antagonist L-798,106, but not by the EP2 or the EP4 receptor antagonists, confirming the involvement of the EP3 receptor subtype in this inhibition of LC neurons. EP1 receptor antagonists were not tested because very low EP1 receptor mRNA expression has been reported in the brainstem (Candelario-Jalil et al., 2005). The fact that the pK_B_ values described for L-798,106 were similar when estimated from the effect of sulprostone or misoprostol (5.8–5.9) as agonists further provides evidence for the involvement of an EP3 receptor subtype. The pK_B_ value for L-798,106 calculated from the effect of PGE_2_ as the agonist was 0.4 log units higher (6.3), suggesting that non-EP3 receptor mechanisms activated by these agonists may interfere in different ways with the regulation of the firing activity of LC neurons by EP3 receptors. In this regard, in the presence of a high concentration of the EP2/EP4 receptor antagonists, the concentration-effect curves for sulprostone and PGE_2_ were shifted to the left but not that of misoprostol. This could be explained by the difference in the phamacological profile of misoprostol for EP3 and EP2 receptors. One could speculate that the EP2 and EP3 receptors physically interact with each other at the postsynaptic site of the LC cell, so that EP2 receptor would prevent the inhibitory effect mediated by EP3 receptor. In the presence of EP2 receptor antagonist, the EP3 receptor would be released and sulprostone and PGE_2_ would elicit greater inhibitory effects onto EP3 receptor. However, in the case of misoprostol, the interaction between receptors would not be as relevant as with the other EP3 receptor agonists due to its lower efficacy than sulprostone at EP3 receptor and the lower selectivity than PGE_2_ for EP2 receptor.

Jones et al. have also reported a higher pK_B_ value for L-798,106 against PGE_2_ effect in guinea-pig vas deferens as compared with the LC herein stated (Jones et al., 2011). However, this discrepancy could be explained by a higher apparent affinity of L-798,106 for EP3 receptors in guinea-pig smooth muscle due to its faster onset of antagonism compared to the brain slice preparation. Thus, highly potent and lipophilic ligands such as L-798,106 have been proposed to underlie slower onset kinetics for EP3 receptors in lipid-containing tissues, which may yield lower pK_B_ values by the forced use of higher antagonist concentrations (Jones et al., 2011).

Therefore, our work demonstrates that the firing activity of LC neurons is inhibited by pharmacologically defined EP3 receptors present within the nucleus. Accordingly, other functional studies at neuronal level have suggested inhibition of brain neurotransmission by EP3 receptors in the LC and other regions. Thus, as abovementioned, whole-cell recordings from mice slices have shown that a single, high concentration of sulprostone (200 nM) causes a hyperpolarization and outward current in neurons of the LC, which results stronger in female animals due to a higher number of EP3 receptors (Mulvey et al., 2018). However, in our study, the electrophysiological responses to a wide range of concentrations of three EP3 receptor agonists (sulprostone, PGE_2_ and misoprostol) were tested both in the absence and in the presence of EP3, EP2 and EP4 receptors antagonists in wild-type rats. We have also described the molecular mechanism involved in the inhibitory responses mediated by the EP3 receptor. In further experiments, it would be interesting to study whether previously described sex-differential electrophysiological responses to EP3 receptor agonists also occur in LC slices from rat. In addition, it is also known that centrally administered EP3 receptor agonists reverse the enhancement of c-fos expression in LC neurons from morphine-dependent rats (Nakagawa et al., 2000). In other brain areas, EP3 receptor activation by PGE_2_ has been reported to weaken glutamatergic transmission in the dorsolateral PAG (Lu et al., 2007), GABAergic transmission in the paraventricular nucleus of the hypothalamus (Khazaeipool et al., 2018) and network activity in the neocortex (Koch et al., 2010). EP3 receptor activation by PGE_2_ also impairs presynaptic long-term potentiation at synapses on pyramidal cells in the hippocampus CA3 (Maingret et al., 2017) and responses to hypercapnia in the chemosensitive parafacial respiratory group (Forsberg et al., 2016). In contrast, activation of postsynaptic EP3 receptors by PGE_2_ depolarizes serotonergic neurons of the dorsal raphe nucleus by a cationic conductance (Momiyama et al., 1996). Finally, EP3 receptors on neurons at supraspinal and spinal levels contribute to regulation of some autonomic functions and nociceptive responses (Su et al., 2008; Drake et al., 2016).

EP3 receptors regulate different sets of G proteins, although the inhibitory G_i/o_ family is the primary transduction mechanism (Markovič et al., 2017). In the LC, G_i/o_ protein-coupled receptors have been shown to open GIRK channels and thereby inhibit neuron activity (Pineda et al., 1997b; Albsoul-Younes et al., 2001; Torrecilla et al., 2002). Herein, the inhibitory effect of the EP3 receptor agonist sulprostone was attenuated by pretreatment with PTX, which blocks G_i/o_ proteins by an ADP-ribosylation of the α subunit (Mangmool and Kurose, 2011). Moreover, sulprostone effect was blunted by barium and SCH-23390, two effective GIRK channel blockers (Kuzhikandathil and Oxford, 2002). Barium and SCH-23390 also increased the firing rate of LC cells, as reported for a disinhibition by these agents in the LC (Li and Putnam, 2013). Taken together, we propose that G_i/o_ protein-gated GIRK channels underlie the inhibitory regulation of LC neurons by EP3 receptors. In cultures of rat hippocampal slices, activation of EP3 receptors augments glutamate-induced excitotoxicity by a mechanism that is ameliorated by pertussis toxin, indicating that G_i/o_ proteins also mediate EP3 receptor effects in CA1 neurons (Ikeda-Matsuo et al., 2010). In sympathetic neurons, GIRK channels are activated by PGE_2_, but the EP receptor subtype has not been yet determined (Ruiz-Velasco and Ikeda, 1998). In addition to the described molecular pathway, we can not completely rule out a presynaptic mechanism that could contribute to the effect of EP3 receptor agonists on the firing rate of LC neurons although this seems very unlikely to occur. EP3 receptor activation leads to an inhibition of GABA release in other brain regions (Ibrahim et al., 1999; Khazaeipool et al., 2018) and LC cells are regulated in an inhibitory manner by GABA_A_ receptor activation (Williams et al., 1991). Therefore, if EP3 receptor agonists had elicited an inhibition of GABA release in LC cells, a stimulation rather than an inhibition of the firing rate would have been observed.

## 5 Conclusion

Our work demonstrates that somatodendritic EP3 receptors suppress the firing activity of LC neurons by opening G_i/o_ protein-coupled GIRK channels. In the entire animal, the inhibitory control of LC noradrenergic neurons by somatodendritic EP3 receptors would be added to the reduction of NA release induced by presynaptic EP3 receptors on nerve terminals as described in the cortex and the hippocampus (Exner and Schlicker, 1995; Günther et al., 2010). The resultant overall decrease in synaptic NA concentrations in the forebrain could explain, among others, the anxiolytic effect of EP3 receptor agonists (Nakagawa et al., 2000), since activation of LC noradrenergic neurons projecting to the prefrontal cortex has been shown to exacerbate anxiety-like behaviors (Hirschberg et al., 2017). In addition to antianxiety actions, EP3 receptors in the LC could play a role in the pathophysiology of pain and inflammatory disorders. Firstly, it has been already shown in arthritis-induced inflammatory models that EP3 receptors in the ventrolateral PAG are involved in hyperalgesia mechanism by facilitating the spinal nociceptive reflex (Drake et al., 2016). We hypothesize that EP3 receptors within the LC may also play a role in the supraspinal regulation of pain *in vivo*, in light of the reported contribution of specific noradrenergic neurons from the LC to the supraspinal inhibitory control of spinal nociception transmission (West et al., 1993; Hickey et al., 2014) and the analgesic effects of opioid agonists (Jongeling et al., 2009). Accordingly, it has been revealed that certain experimental models that generate an activation of LC neurons via prostanoid synthesis are also capable of transforming normally innocuous stimuli into allodynia-like nociceptive responses (Hall et al., 1999). Furthermore, CFA-induced persistent inflammatory pain attenuates the antinociceptive effect of the µ opioid receptor agonist DAMGO applied into the LC, confirming the relevance of inflammatory factors in the mutual regulation of nociception and the LC (Jongeling et al., 2009). Regarding neuroinflammatory states, systemic infusion of pyrogenic agents (i.e., LPS) causes an overactivation of LC neurons through central PGE_2_ synthesis (Dallaporta et al., 2007), whereas central and systemic administrations of LPS or PGE_2_ induce febrile responses through both the LC and the EP3 receptor (Ushikubi et al., 1998; Almeida et al., 2004). Finally, animal models of Alzheimer’s disease have shown an overexpression of inhibitory EP3 receptors around amyloid plaques in LC-projecting brain areas (Shi et al., 2012). This change may have a protective role, since amyloid-β deposition and proinflammatory cytokine production in the hippocampus from these animals are both aggravated by lesions of the LC (Heneka et al., 2010; Bharani et al., 2017). However, chronic neuroinflammatory states as that caused by prolonged, central (i.c.v.) LPS administration also enhances the production of inflammation factors within the LC, which leads to a disruption of Ca^2+^ channel-dependent neuron pacemaking activity and thereby a neurodegenerative loss of LC noradrenergic cells (Hopp et al., 2015); this change has been associated with the maladaptive behavioral agitation phenotype found in Alzheimer´s disease (Hopp et al., 2015). All these data point to the interest of prostanoid EP3 receptors in the LC as novel pharmacological targets for neuropsychiatric disorders.

## Data Availability

The raw data supporting the conclusion of this article will be made available by the authors, without undue reservation.

## Abbreviations

aCSF, artificial cerebrospinal fluid; FR, firing rate; GIRK, inwardly rectifying potassium channel; LC, locus coeruleus; ME, [Met]enkephalin; NA, noradrenaline; NSAID, Nonsteroidal anti-inflammatory drugs; PAG, periaqueductal gray; PTX, pertussis toxin; PGE_2_, prostaglandin E_2_; PGE_1_, prostaglandin E_1_.

## References

[B1] Abramovitz M. Adam M. Boie Y. Carrière M. C. Denis D. Godbout C. (2000). The utilization of recombinant prostanoid receptors to determine the affinities and selectivities of prostaglandins and related analogs. Biochim. Biophys. Acta - Mol. Cell Biol. Lipids. 1483, 285–293. 10.1016/S1388-1981(99)00164-X 10634944

[B2] Albsoul-Younes A. M. Sternweis P. M. Zhao P. Nakata H. Nakajima Y. Kozasa T. (2001). Interaction sites of the G protein beta subunit with brain G protein-coupled inward rectifier K+ channel. J. Biol. Chem. 276, 12712–12717. 10.1074/jbc.M011231200 11278861

[B3] Almeida M. C. a Steiner A. Coimbra N. C. Branco L. G. S. (2004). Thermoeffector neuronal pathways in fever: a study in rats showing a new role of the locus coeruleus. J. Physiol. 558, 283–294. 10.1113/jphysiol.2004.066654 15146040 PMC1664907

[B4] Bharani K. L. Derex R. Granholm A. C. Ledreux A. (2017). A noradrenergic lesion aggravates the effects of systemic inflammation on the hippocampus of aged rats. PLoS One 12, 01898211–e189920. 10.1371/journal.pone.0189821 PMC5736222 29261743

[B5] Candelario-Jalil E. Slawik H. Ridelis I. Waschbisch A. Akundi R. S. Hüll M. (2005). Regional distribution of the prostaglandin E2 receptor EP1 in the rat brain: accumulation in Purkinje cells of the cerebellum. J. Mol. Neurosci. 27, 303–310. 10.1385/JMN:27:3:303 16280600

[B6] Chee M. J. Price C. J. a Statnick M. Colmers W. F. (2011). Nociceptin/orphanin FQ suppresses the excitability of neurons in the ventromedial nucleus of the hypothalamus. J. Physiol. 589, 3103–3114. 10.1113/jphysiol.2011.208819 21502286 PMC3145927

[B7] Chessell I. P. Black M. D. Feniuk W. Humphrey P. P. A. (1996). Operational characteristics of somatostatin receptors mediating inhibitory actions on rat locus coeruleus neurones. Br. J. Pharmacol. 117, 1673–1678. 10.1111/j.1476-5381.1996.tb15338.x 8732275 PMC1909561

[B8] Curtis M. J. Alexander S. Cirino G. Docherty J. R. George C. H. Giembycz M. A. (2018). Experimental design and analysis and their reporting II: updated and simplified guidance for authors and peer reviewers. Br. J. Pharmacol. 175, 987–993. 10.1111/bph.14153 29520785 PMC5843711

[B9] Dallaporta M. Pecchi E. Jacques C. Berenbaum F. Jean A. Thirion S. (2007). c-Fos immunoreactivity induced by intraperitoneal LPS administration is reduced in the brain of mice lacking the microsomal prostaglandin E synthase-1 (mPGES-1). Brain. Behav. Immun. 21, 1109–1121. 10.1016/j.bbi.2007.05.003 17604949

[B10] Drake R. A. R. Leith J. L. Almahasneh F. Martindale J. Wilson A. W. Lumb B. (2016). Periaqueductal grey EP3 receptors facilitate spinal nociception in arthritic secondary hypersensitivity. J. Neurosci. 36, 9026–9040. 10.1523/JNEUROSCI.4393-15.2016 27581447 PMC5005717

[B11] Ek M. Arias C. Sawchenko P. Ericsson-Dahlstrand A. (2000). Distribution of the EP3 prostaglandin E2 receptor subtype in the rat brain: relationship to sites of interleukin-1 - induced cellular responsiveness. J. Comp. Neurol. 428, 5–20. 10.1002/1096-9861(20001204)428:1<5::AID-CNE2>3.0.CO;2-M 11058221

[B12] Exner H. J. Schlicker E. (1995). Prostanoid receptors of the EP3 subtype mediate the inhibitory effect of prostaglandin E2 on noradrenaline release in the mouse brain cortex. Naunyn. Schmiedeb. Arch. Pharmacol. 351, 46–52. 10.1007/BF00169063 7715741

[B13] Forsberg D. Horn Z. Tserga E. Smedler E. Silberberg G. Shvarev Y. (2016). CO2-evoked release of PGE2 modulates sighs and inspiration as demonstrated in brainstem organotypic culture. eLife 5, 141700–e14241. 10.7554/eLife.14170 PMC4974055 27377173

[B14] Forselles K. J. Root J. Clarke T. Davey D. Aughton K. Dack K. (2011). *In vitro* and *in vivo* characterization of PF-04418948, a novel, potent and selective prostaglandin EP₂ receptor antagonist. Br. J. Pharmacol. 164, 1847–1856. 10.1111/j.1476-5381.2011.01495.x 21595651 PMC3246710

[B15] Günther J. Schulte K. Wenzel D. Malinowska B. Schlicker E. (2010). Prostaglandins of the E series inhibit monoamine release via EP3 receptors: proof with the competitive EP3 receptor antagonist L-826,266. Naunyn. Schmiedeb. Arch. Pharmacol. 381, 21–31. 10.1007/s00210-009-0478-9 20012265

[B16] Hall S. Milne B. Loomis C. (1999). Spinal action of ketorolac, S(+)- and R(-)-ibuprofen on non-noxious activation of the catechol oxidation in the rat locus coeruleus: evidence for a central role of prostaglandins in the strychnine model of allodynia. Anesthesiology 90, 165–173. 10.1097/00000542-199901000-00022 9915325

[B17] Heneka M. T. Nadrigny F. Regen T. Martinez-Hernandez A. Dumitrescu-Ozimek L. Terwel D. (2010). Locus ceruleus controls Alzheimer’s disease pathology by modulating microglial functions through norepinephrine. Proc. Natl. Acad. Sci. 107, 6058–6063. 10.1073/pnas.0909586107 20231476 PMC2851853

[B18] Hétu P.-O. Riendeau D. (2005). Cyclo-oxygenase-2 contributes to constitutive prostanoid production in rat kidney and brain. Biochem. J. 391, 561–566. 10.1042/BJ20050451 16008526 PMC1276956

[B19] Hickey L. Li Y. Fyson S. J. Watson T. C. Perrins R. Hewinson J. (2014). Optoactivation of locus ceruleus neurons evokes bidirectional changes in thermal nociception in rats. J. Neurosci. 34, 4148–4160. 10.1523/JNEUROSCI.4835-13.2014 24647936 PMC3960461

[B20] Hirschberg S. Li Y. Randall A. Kremer E. J. Pickering A. E. (2017). Functional dichotomy in spinal-vs prefrontal-projecting locus coeruleus modules splits descending noradrenergic analgesia from ascending aversion and anxiety in rats. eLife 6, e29808–e29826. 10.7554/eLife.29808 29027903 PMC5653237

[B21] Hopp S. C. Royer S. E. D'Angelo H. M. Kaercher R. M. Fisher D. A. Wenk G. L. (2015). Differential neuroprotective and anti-inflammatory effects of L-type voltage dependent calcium channel and ryanodine receptor antagonists in the substantia nigra and locus coeruleus. J. Neuroimmune Pharmacol. 10, 35–44. 10.1007/s11481-014-9568-7 25318607 PMC4336597

[B22] Ibrahim N. Shibuya I. Kabashima N. Sutarmo S. V. Ueta Y. Yamashita H. (1999). Prostaglandin E2 inhibits spontaneous inhibitory postsynaptic currents in rat supraoptic neurones via presynaptic EP receptors. J. Neuroendocrinol. 11, 879–886. 10.1046/j.1365-2826.1999.00404.x 10520139

[B23] Ikeda-Matsuo Y. Tanji H. Ota a. Hirayama Y. Uematsu S. Akira S. (2010). Microsomal prostaglandin E synthase-1 contributes to ischaemic excitotoxicity through prostaglandin E2 EP3 receptors. Br. J. Pharmacol. 160, 847–859. 10.1111/j.1476-5381.2010.00711.x 20590584 PMC2935993

[B24] Jones R. L. Qian Y. M. Chan K. M. Yim a P. (1998). Characterization of a prostanoid EP3-receptor in Guinea-pig aorta: partial agonist action of the non-prostanoid ONO-AP-324. Br. J. Pharmacol. 125, 1288–1296. 10.1038/sj.bjp.0702189 9863659 PMC1565698

[B25] Jones R. L. Woodward D. F. Wang J. W. Clark R. L. (2011). Roles of affinity and lipophilicity in the slow kinetics of prostanoid receptor antagonists on isolated smooth muscle preparations. Br. J. Pharmacol. 162, 863–879. 10.1111/j.1476-5381.2010.01087.x 20973775 PMC3042197

[B26] Jongeling A. C. Jouns E. M. Murphy A. Z. Hammond D. L. (2009). Persistent inflammatory pain decreases the antinociceptive effects of the mu opioid receptor agonist DAMGO in the locus coeruleus of male rats. Neuropharmacology 56, 1017–1026. 10.1016/j.neuropharm.2009.02.005 19265713 PMC2680457

[B27] Kaufmann W. E. Worley P. F. Pegg J. Bremer M. Isakson P. (1996). COX-2, a synaptically induced enzyme, is expressed by excitatory neurons at postsynaptic sites in rat cerebral cortex. Proc. Natl. Acad. Sci. U. S. A. 93, 2317–2321. 10.1073/pnas.93.6.2317 8637870 PMC39793

[B28] Khazaeipool Z. Wiederman M. Inoue W. (2018). Prostaglandin E2 depresses GABA release onto parvocellular neuroendocrine neurones in the paraventricular nucleus of the hypothalamus via presynaptic receptors. J. Neuroendocrinol. 30, 126388–e12710. 10.1111/jne.12638 30084511

[B29] Kilkenny C. Browne W. J. Cuthill I. C. Emerson M. Altman D. G. (2010). Improving bioscience research reporting: the ARRIVE guidelines for reporting animal research. PLOS Biol. 8, e1000412–e1000415. 10.1371/journal.pbio.1000412 20613859 PMC2893951

[B30] Koch H. Huh S.-E. Elsen F. P. Carroll M. S. Hodge R. D. Bedogni F. (2010). Prostaglandin E2-induced synaptic plasticity in neocortical networks of organotypic slice cultures. J. Neurosci. 30, 11678–11687. 10.1523/JNEUROSCI.4665-09.2010 20810888 PMC3842478

[B59] Kuzhikandathil E. V. Oxford G. S. (2002). Classic D1 dopamine receptor AntagonistR-(+)-7-chloro-8-hydroxy-3-methyl-1-phenyl-2,3,4,5-tetrahydro-1H-3-benzazepine hydrochloride (SCH23390) directly inhibits G protein-coupled inwardly rectifying potassium channels. Mol. Pharmacol. 62, 119–126. 10.1124/mol.62.1.119 12065762

[B31] Lazareno S. Birdsall N. J. M. (1993). Estimation of competitive antagonist affinity from functional inhibition curves using the Gaddum Schild and Cheng-Prusoff equations. Br. J. Pharmacol. 109, 1110–1119. 10.1111/j.1476-5381.1993.tb13737.x 8401922 PMC2175764

[B32] Li K.-Y. Putnam R. W. (2013). Transient outwardly rectifying A currents are involved in the firing rate response to altered CO2 in chemosensitive locus coeruleus neurons from neonatal rats. Am. J. Physiol. Regul. Integr. Comp. Physiol. 305, R780–R792. 10.1152/ajpregu.00029.2013 23948777 PMC3798795

[B33] Lu J. Xing J. Li J. (2007). Prostaglandin E2 (PGE2) inhibits glutamatergic synaptic transmission in dorsolateral periaqueductal gray (dl-PAG). Brain Res. 1162, 38–47. 10.1016/j.brainres.2007.06.004 17612511 PMC2030489

[B34] Machwate M. Harada S. Leu C. T. Seedor G. Labelle M. Gallant M. (2001). Prostaglandin receptor EP(4) mediates the bone anabolic effects of PGE(2). Mol. Pharmacol. 60, 36–41. 10.1124/mol.60.1.36 11408598

[B35] Maingret V. Barthet G. Deforges S. Jiang N. Mulle C. Amédée T. (2017). PGE2-EP3 signaling pathway impairs hippocampal presynaptic long-term plasticity in a mouse model of Alzheimer’s disease. Neurobiol. Aging. 50, 13–24. 10.1016/j.neurobiolaging.2016.10.012 27837675

[B36] Mangmool S. Kurose H. (2011). Gi/o protein-dependent and -independent actions of pertussis toxin (ptx). Toxins (Basel) 3, 884–899. 10.3390/toxins3070884 22069745 PMC3202852

[B37] Markovič T. Jakopin Ž. Dolenc M. S. Mlinarič-Raščan I. (2017). Structural features of subtype-selective EP receptor modulators. Drug Discov. Today. 22, 57–71. 10.1016/j.drudis.2016.08.003 27506873

[B38] Medrano M. C. Santamarta M. T. Pablos P. Aira Z. Buesa I. Azkue J. J. (2017). Characterization of functional μ opioid receptor turnover in rat locus coeruleus: an electrophysiological and immunocytochemical study. Br. J. Pharmacol. 174, 2758–2772. 10.1111/bph.13901 28589556 PMC5522999

[B39] Mendiguren A. Pineda J. (2004). Cannabinoids enhance N-methyl-D-aspartate-induced excitation of locus coeruleus neurons by CB1 receptors in rat brain slices. Neurosci. Lett. 363, 1–5. 10.1016/j.neulet.2004.02.073 15157983

[B40] Mendiguren A. Pineda J. (2007). CB1 cannabinoid receptors inhibit the glutamatergic component of KCl-evoked excitation of locus coeruleus neurons in rat brain slices. Neuropharmacology 52, 617–625. 10.1016/j.neuropharm.2006.09.004 17070872

[B41] Momiyama T. Todo N. Sugimoto Y. Ichikawa A. Narumiya S. (1996). Membrane depolarization by activation of prostaglandin E receptor EP 3 subtype of putative serotonergic neurons in the dorsal raphe nucleus of the rat. Naunyn. Schmiedeb. Arch. Pharmacol. 353, 377–381. 10.1007/BF00261433 8935703

[B42] Mulvey B. Bhatti D. L. Gyawali S. Lake A. M. Kriaucionis S. Ford C. P. (2018). Molecular and functional sex differences of noradrenergic neurons in the mouse locus coeruleus. Cell Rep. 23, 2225–2235. 10.1016/j.celrep.2018.04.054 29791834 PMC6070358

[B43] Nakagawa T. Masuda T. Watanabe T. Minami M. Satoh M. (2000). Possible involvement of the locus coeruleus in inhibition by prostanoid EP(3) receptor-selective agonists of morphine withdrawal syndrome in rats. Eur. J. Pharmacol. 390, 257–266. 10.1016/s0014-2999(99)00901-2 10708732

[B44] Nakamura K. Li Y. Q. Kaneko T. Katoh H. Negishi M. (2001). Prostaglandin EP3 receptor protein in serotonin and catecholamine cell groups: a double immunofluorescence study in the rat brain. Neuroscience 103, 763–775. 10.1016/S0306-4522(01)00027-6 11274793

[B66] Paxinos G. Watson C. (2009). The rat brain in stereotaxic coordinates compact. 6th Edn. London, Burlington: San Diego: Academic Press.

[B45] Pablos P. Mendiguren A. Pineda J. (2015). Contribution of nitric oxide-dependent guanylate cyclase and reactive oxygen species signaling pathways to desensitization of μ-opioid receptors in the rat locus coeruleus. Neuropharmacology 99, 422–431. 10.1016/j.neuropharm.2015.08.004 26254861

[B46] Pineda J. Aghajanian G. K. (1997). Carbon dioxide regulates the tonic activity of locus coeruleus neurons by modulating a proton- and polyamine-sensitive inward rectifier potassium current. Neuroscience 77, 723–743. 10.1016/s0306-4522(96)00485-x 9070748

[B47] Pineda J. Kogan J. H. Aghajanian G. K. (1996). Nitric oxide and carbon monoxide activate locus coeruleus neurons through a cGMP-dependent protein kinase: involvement of a nonselective cationic channel. J. Neurosci. 16, 1389–1399. 10.1523/JNEUROSCI.16-04-01389.1996 8778290 PMC6578554

[B48] Pineda J. Ruiz-Ortega J. A. Ugedo L. (1997b). Receptor reserve and turnover of alpha-2 adrenoceptors that mediate the clonidine-induced inhibition of rat locus coeruleus neurons *in vivo* . J. Pharmacol. Exp. Ther. 281, 690–698. 9152374

[B49] Pineda J. Ugedo L. a García-Sevilla J. (1997a). Enhanced alpha2A-autoreceptor reserve for clonidine induced by reserpine and cholinomimetic agents in the rat vas deferens. Br. J. Pharmacol. 122, 833–840. 10.1038/sj.bjp.0701455 9384498 PMC1565011

[B50] Ruiz-Velasco V. Ikeda S. R. (1998). Heterologous expression and coupling of G protein-gated inwardly rectifying K+ channels in adult rat sympathetic neurons. J. Physiol. 513, 761–773. 10.1111/j.1469-7793.1998.761ba.x 9824716 PMC2231322

[B51] Säfholm J. Dahlén S.-E. Delin I. Maxey K. Stark K. Cardell L.-O. (2013). PGE 2 maintains the tone of the Guinea pig trachea through a balance between activation of contractile EP 1 receptors and relaxant EP 2 receptors. Br. J. Pharmacol. 168, 794–806. 10.1111/j.1476-5381.2012.02189.x 22934927 PMC3631371

[B52] Sang N. Zhang J. Marcheselli V. Bazan N. G. Chen C. (2005). Postsynaptically synthesized prostaglandin E2 (PGE2) modulates hippocampal synaptic transmission via a presynaptic PGE2 EP2 receptor. J. Neurosci. 25, 9858–9870. 10.1523/JNEUROSCI.2392-05.2005 16251433 PMC6725559

[B53] Shi J. Wang Q. Johansson J. U. Liang X. Breyer R. M. Montine T. J. (2012). Inflammatory prostaglandin E2 signaling in a mouse model of Alzheimer disease. Ann. Neurol. 72, 788–798. 10.1002/ana.23677 22915243 PMC3509238

[B54] Su X. a Leon L. Wu C. W. Morrow D. M. Jaworski J.-P. Hieble J. P. (2008). Modulation of bladder function by prostaglandin EP3 receptors in the central nervous system. Am. J. Physiol. Ren. Physiol. 295, F984–F994. 10.1152/ajprenal.90373.2008 18632791

[B55] Sugimoto Y. Shigemoto R. Namba T. Negishi M. Mizuno N. Narumiya S. (1994). Distribution of the messenger RNA for the prostaglandin E receptor subtype EP3 in the mouse nervous.system. Neuroscience 62, 919–928. 10.1016/0306-4522(94)90483-9 7870313

[B56] Torrecilla M. Marker C. L. Cintora S. C. Stoffel M. Williams J. T. Wickman K., G-protein-gated potassium channels containing Kir3.2 and Kir3.3 subunits mediate the acute inhibitory effects of opioids on locus ceruleus neurons, J. Neurosci. 22 (2002) 4328–4334. 20026414. 10.1523/JNEUROSCI.22-11-04328.2002 12040038 PMC6758804

[B57] Ushikubi F. Segi E. Sugimoto Y. Murata T. Matsuoka T. Kobayashi T. (1998). Impaired febrile response in mice lacking the prostaglandin E receptor subtype EP3. Nature 395, 281–284. 10.1038/26233 9751056

[B58] Van Bockstaele E. J. (1998). Morphological substrates underlying opioid, epinephrine and gamma-aminobutyric acid inhibitory actions in the rat locus coeruleus. Brain Res. Bull. 47 (1), 1–15. 10.1016/s0361-9230(98)00062-8 9766384

[B60] Waud D. R. Lee Son S. Waud B. E. (1978). Kinekic and empirical analysis of dose-response curves illustrated with a cardiac example. Life Sci. 22, 1275–1286. 10.1016/0024-3205(78)90096-6 661504

[B61] West W. L. Yeomans D. C. Proudfit H. K. (1993). The function of noradrenergic neurons in mediating antinociception induced by electrical stimulation of the locus coeruleus in two different sources of Sprague-Dawley rats. Brain Res. 626, 127–135. 10.1016/0006-8993(93)90571-4 7904225

[B62] Williams J. T. Bobker D. H. Harris G. C. (1991). Synaptic potentials in locus coeruleus neurons in brain slices. Prog. Brain Res. 88, 167–172. 10.1016/s0079-6123(08)63806-6 1726025

[B63] Williams J. T. North R. A. Shefner S. A. Nishi S. Egan T. M. (1984). Membrane properties of rat locus coeruleus neurones. Neuroscience 13 (1), 137–156. 10.1016/0306-4522(84)90265-3 6493483

[B64] Yamaguchi N. Okada S. (2009). Cyclooxygenase-1 and -2 in spinally projecting neurons are involved in CRF-induced sympathetic activation. Auton. Neurosci. Basic Clin. 151, 82–89. 10.1016/j.autneu.2009.06.009 19632905

[B65] Zhang J. Rivest S. (1999). Distribution, regulation and colocalization of the genes encoding the EP 2 - and EP 4 -PGE 2 receptors in the rat brain and neuronal responses to systemic inflammation. Eur. J. Neurosci. 11, 2651–2668. 10.1046/j.1460-9568.1999.00682.x 10457163

